# The Role of NOTCH Pathway Genes in the Inherited Susceptibility to Aortic Stenosis

**DOI:** 10.3390/jcdd11070226

**Published:** 2024-07-17

**Authors:** Olga Irtyuga, Rostislav Skitchenko, Mary Babakekhyan, Dmitrii Usoltsev, Svetlana Tarnovskaya, Anna Malashicheva, Yulya Fomicheva, Oksana Rotar, Olga Moiseeva, Ulyana Shadrina, Mykyta Artomov, Anna Kostareva, Evgeny Shlyakhto

**Affiliations:** 1 Almazov National Medical Research Centre, 197341 Saint-Petersburg, Russia; rost20151995@gmail.com (R.S.); babakekhyan_mv@almazovcentre.ru (M.B.); dmitrii.usoltsev@nationwidechildrens.org (D.U.); tarnovskaya_si@almazovcentre.ru (S.T.); malashicheva_ab@almazovcentre.ru (A.M.); fomicheva_yuv@almazovcentre.ru (Y.F.); rotari_oxana@mail.ru (O.R.); moiseeva_om@almazovcentre.ru (O.M.); shadrina_um@almazovcentre.ru (U.S.); mykyta.artomov@nationwidechildrens.org (M.A.); akostareva@hotmail.com (A.K.); e.shlyakhto@almazovcentre.ru (E.S.); 2 Steve and Cindy Rasmussen Institute for Genomic Medicine, Nationwide Children’s Hospital, Columbus, OH 43205, USA; 3 Department of Pediatrics, The Ohio State University College of Medicine, Columbus, OH 43205, USA; 4 Department of Women’s and Children’s Health and Centre for Molecular Medicine, Karolinska Institute, 17176 Stockholm, Sweden

**Keywords:** aortic stenosis, calcified aortic valve disease, signal transduction, NOTCH-signaling pathway

## Abstract

The NOTCH-signaling pathway is responsible for intercellular interactions and cell fate commitment. Recently, NOTCH pathway genes were demonstrated to play an important role in aortic valve development, leading to an increased calcified aortic valve disease (CAVD) later in life. Here, we further investigate the association between genetic variants in the NOTCH pathway genes and aortic stenosis in a case–control study of 90 CAVD cases and 4723 controls using target panel sequencing of full-length 20 genes from a NOTCH-related pathway (*DVL2*, *DTX2*, *MFNG*, *NUMBL*, *LFNG*, *DVL1*, *DTX4*, *APH1A*, *DTX1*, *APH1B*, *NOTCH1*, *ADAM17*, *DVL3*, *NCSTN*, *DTX3L*, *ILK*, *RFNG*, *DTX3*, *NOTCH4*, *PSENEN*). We identified a common intronic variant in *NOTCH1*, protecting against CAVD development (rs3812603), as well as several rare and unique new variants in NOTCH-pathway genes (*DTX4*, *NOTCH1*, *DTX1*, *DVL2*, *NOTCH1*, *DTX3L*, *DVL3*), with a prominent effect of the protein structure and function.

## 1. Introduction

Aortic valve stenosis (AS) is one of the most common valvular heart diseases in developed countries [1]. The results of several epidemiological studies demonstrated that the incidence of AS increases with age and the prevalence of AS among people over 75 years is 12.4% (from 6.6% to 18.2%), and for severe forms of AS, it ranges from 1.1% to 5.7% [2,3]. Currently, the most common cause of aortic stenosis is calcification of the tricuspid (TAV) and bicuspid (BAV) aortic valves due to degenerative calcific aortic valve disease (CAVD), with other causes being significantly less common [4]. BAV is one of the most frequently diagnosed congenital heart defects, occurring in 1–2% of the population [5,6]. Due to impaired vale architecture and increased hemodynamical stress, BAV patients are more prone to aortic valve calcification [5]. The clinical importance of CAVD is strengthened by the limited number of therapeutical approaches and absence of effective targeted drug, which can delay or stop the progression of aortic stenosis [7].

Several signaling cascades important for embryonic heart development have been shown to play an important role in cardiovascular pathology in an adult life. Among those, NOTCH-signaling was described as one of the key contributors of aorta and aortic valve pathologies [8,9,10]. The NOTCH pathway is one of the main intercellular signaling mechanisms that mediates the interaction and communication between neighboring cells. NOTCH-signaling links the fate of one cell to that of surrounding cells through physical interaction between the NOTCH receptor and membrane-bound ligands expressed in the nearby cell. The outcome of NOTCH-signaling is highly pleiotropic, strictly dependent on the cellular context and can affect cell differentiation, proliferation and apoptosis [11]. An alteration in NOTCH1-signaling is associated with a wide range of diseases, including various types of malignancies and developmental disorders [8].

In the cardiovascular system, NOTCH signaling is involved in angiogenesis, early cardiomyocyte differentiation [12], ventricular trabeculation [13], cardiac valve formation and [14] outflow tract development [15]. Consequently, *NOTCH1* variants were identified as a cause of congenital BAV, CAVD and aortic aneurhysma; the causative role of *NOTCH1* and several other genes linked to the *NOTCH1* signaling cascade, such as *DLL1* and *JAGGED1*, with congenital cardiovascular pathologies has been reported [16].

To further decipher the association of genetic landscape in NOTCH-signaling genes with CAVD, we sequenced coding and non-coding regions of 20 genes involved in the NOTCH-signaling cascade (*DVL2*, *DTX2*, *MFNG*, *NUMBL*, *LFNG*, *DVL1*, *DTX4*, *APH1A*, *DTX1*, *APH1B*, *NOTCH1*, *ADAM17*, *DVL3*, *NCSTN*, *DTX3L*, *ILK*, *RFNG*, *DTX3*, *NOTCH4*, *PSENEN*) in a cohort of 90 patients diagnosed with AS. With this approach, we identified a potentially protective common genetic variant rs3812603 in *NOTCH1* and risk variant rs73185723 in *DVL3*, as well as several rare and new variants of unknown significance with potential impact on the development of CAVD.

## 2. Materials and Methods

### 2.1. Ethics Statement

This study was performed according to the Declaration of Helsinki, and approval was obtained from Almazov National Medical Research Centre Ethical Committee (Saint-Petersburg, Russian Federation) before the initiation of the study. Written form informed consent was obtained from all participating patients (Protocol № 24 dated 23 March 2020).

### 2.2. Discovery Cohort Description

Of the total database of patients with AS, 90 patients with severe AS were randomly selected who were surgically treated at Almazov National Medical Research Center, St. Petersburg, Russian Federation between 2010 and 2018. The type of aortic valve morphology was confirmed intraoperatively.

Inclusion criteria:

- Patients with AS of TAV and BAV due to CAVD.
- Patient with AS and Vmax at the aortic valve more than 4.0 m/s and effective orifice area less than 1 cm^2^.
- Patients with intraoperatively confirmed BAV or TAV.
- Patients with genotype–phenotype correlation analysis AS.
- Consent of the patient to be included in the study.

Exclusion criteria:

- Patient with AS and Vmax at the aortic valve that was less than 4.0 m/s and effective orifice area that was more than 1 cm^2^.
- Patients with known infective endocarditis and rheumatic disease.
- Patients with connective tissue disorder (like Marfan) and/or positive family history of aortic valve disease or aortic aneurysm.
- Refusal of the patient to be included in the study.

### 2.3. Control Cohort Description

The control group included 4723 DNA samples that were collected within a framework of the national epidemiological study—ESSE RF. The control subjects were not ascertained for the aortic stenosis and include a random snapshot of the population aged 18–64 years old. The control group was genotyped using the FinnGen Axiom array and genotypes were imputed using the HRC reference panel. Genetic and clinical data collection and analysis for the control cohort were earlier described [17].

### 2.4. Clinical Assessment and Echocardiography

All patients in this study underwent a comprehensive two-dimensional and Doppler transthoracic ECHO using the Vivid 7.0 system (General Electric, Chicago, IL, USA) according to the current guidelines [18]. The criteria for severity of aortic valve stenosis included aortic valve area (AVA, cm^2^), calculated using the continuity equation, AVA indexed for body surface area (AVA/BSA, cm^2^/m^2^), mean transvalvular pressure gradient and peak aortic jet velocity (Vmax). A patient was included in the study group if Vmax at the aortic valve was more than 4.0 m/s [18]. Diagnosis of BAV was based on short-axis imaging of the aortic valve, demonstrating the existence of only 2 commissures delimiting only 2 aortic valve cusps.

### 2.5. NOTCH Panel Sequencing and Variant Validation

The Haloplex Target Enrichment System (Agilent, Waldbronn, Germany) for Illumina MiSeq instrument (Illumina, San Diego, CA, USA) included 20 genes participating in NOTCH-signaling cascade. Sequencing protocol, library preparation and variant validation were performed according to standard protocol as earlier described [19]. Alignment, data processing and variant calling were performed according to GATK BestPractice recommendations (Broad Institute, Cambridge, MA, USA) using hg38 human genome reference. Variant annotation was performed using ANNOVAR (Philadelphia, PA, USA) and the assessment of rare and newly identified variants was performed according to ACMG guidelines [20]. All rare and newly identified variants were confirmed using Sanger sequencing.

For the case–control analysis, the following quality filtration was applied: (1) DP ≥ 10; (2) GQ ≥ 20; (3) allele balance filtration; (4) Hardy–Weinberg equilibrium *p* > 10^−4^; (5) variant call rate ≥ 0.8; and (6) sample call rate ≥ 0.8. For a case–control analysis of common variants (MAF > 0.01), we kept 284 variants that passed quality filtration both in the case and control cohorts. The identification of significant and considerable genetic variants was carried out using expression quantitative trait loci (eQTL) and the Genotype-Tissue Ex-pression (GTEx).

### 2.6. Protein Structural Modeling

The protein sequences and domain organization were obtained from the Uniprot database [21]. Functional prediction and annotation of missense variants in the human genes were assessed based on five prediction scores (REVEL, VEST4, ClinPred, SIFT, Mutation Taster, Provean) obtained from the dbNSFPv4.4 database [22]. Each domain in a given protein was numbered according to its order of appearance in a protein structure. Related protein sequences from other organisms were identified by BLASTP [23] searches against the UniProt/SwissProt database using the E-value threshold of 0.001. Multiple sequence alignments for individual domains were calculated by T-Coffee [24] and visualized in Jalview 2.8.2 [25]. We calculated the position-specific conservation score (Cs), which varies between 0 (is not conserved) and 5 (is conserved). Conservation scores of identical positions are set to 11.

Known 3D structures of protein domains were extracted from the Protein Data Bank [26]. If the 3D structure was absent in the database, we chose Alphafold2 protein structure obtained from the Uniprot database. In silico mutagenesis and visualization were conducted using the PyMol v2.5.0 software.

## 3. Results

The study group included 90 CAVD patients—59 with BAV and 31 patients with TAV (Table 1). The BAV subgroup was significantly younger than the subgroup of TAV patients (*p* = 0.00004). The levels of maximum systolic blood pressure (Systolic BP) and maximum diastolic blood pressure (Diastolic BP) were higher in the TAV subgroup (*p* = 0.02). In addition, higher levels of C-reactive protein (*p* = 0.02) were found in the group of patients with TAV. Patients with BAV had higher levels of total cholesterol (*p* = 0.03). No other significant demographic and clinical differences were noted between the BAV and TAV patients.

Sequencing analysis of 20 NOTCH pathway-related genes was performed using NGS target panel and resulted in the identification of coding and non-coding rare and common genetic variants. As a first step, we performed an association analysis comparing the data with populational cohort data previously obtained on 4723 subjects. An association analysis revealed a common variant rs3812603 in the intron of *NOTCH1* (NC_000016.10:g.136508456T>C, rs3812603) underrepresented in the study group (atrial fibrillation (AF) in CAVD group 0.42, AF in controls 0.59, *p*-value = 1.90 × 10^−5^; β = −0.0112) and a common variant in intron of *DVL3* (NC_000003.12:g.184168569C>T, rs73185723) overrepresented in CAVD patients (AF in CAVD group 0.22, AF in controls 0.12, *p*-value = 1.49 × 10^−4^; β = 0.0149). Analysis of eQTL using GTEx data showed that NC_000016.10:g.136508456T>C and NC_000003.12:g.184168569C>T were significantly associated with increased expression in aortic tissue (*p*-value = 3.8 × 10^−5^ and *p*-value = 8.3 × 10^−6^, respectively,).

The coverage data allowed us to perform deeper genotype–phenotype correlation analysis on 76 out of 90 patients; 14 patients were excluded from the analysis due to the low number of reads covering the chr9:136508456 region. Detailed clinical analysis of 76 patients depending on NOTCH1 intronic rs3812603 genotype reveled an association of T-allele with lower systolic blood pressure (*p* = 0.02), meaning that carriers of T-allele develop CAVD on a lower hypertensive level (Table 2), although this was not significant after multiple hypothesis correction. No other differences in clinical parameters were noted between the carriers of T and C-alleles.

In a sub-cohort of BAV patients, we identified nominal association of T allele with smaller aortic size at sinus level (*p* = 0.02, Table 3).

In the TAV-only patient group, the genotype–phenotype association analysis was not performed due to the low number of patients included (n = 31). To sum up, genotype association analysis performed on genetic variants from the entire regions of 20 NOTCH-signaling-related genes identified that allele T rs3812603 is overrepresented in the CAVD group and is associated with lower systolic blood pressure and a small diameter of the aortic sinus.

In patients carrying the DLV3 rs73185723 variant, there were no significant differences among the groups of patients with T- and C-alleles in characteristics such as gender, age, aortic size, CHF and CHD. However, the clinical analysis of patients depending on the rs73185723 genotype revealed nominal associations of C-allele with lower systolic (*p* = 0.007) and diastolic (*p* = 0.04) blood pressure, meaning that carriers of C-allele develop CAVD on a lower hypertensive level (Table 4).

However, when restricting the analysis only to BAV patients, T-allele was associated with higher blood pressure levels, both systolic (*p* = 0.005) and diastolic (*p* = 0.02). No other differences in clinical parameters were noted between the carriers of T and C-alleles (Table 5).

As a next step, sequencing analysis of 20 NOTCH-pathway-related genes aimed to identify unique and rare (<1:10,000) coding genetic variants for their possible impact on the development of aortic valve calcification and CAVD. We identified seven rare coding variants in six patients, including two previously unreported variants and one LOF (Table 6).

**Figure 1:**
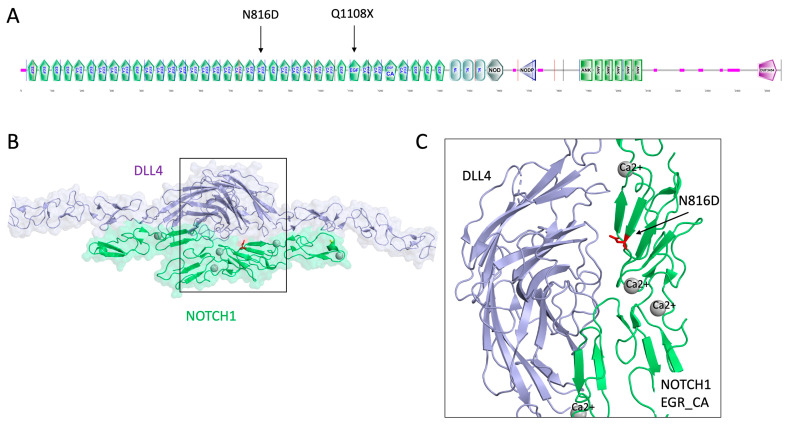
Structure of NOTCH1 EGF CA domain. (**A**) Domain organization of the human Notch1 protein sequence performed using the Uniprot database. Variants p.Asn816Asp and p.Gln1108Ter, are mapped to the protein structure. (**B**) Three-dimensional structure of EGF modules of the NOTCH extracellular domain bound to DLL4 [29]. (**C**) Interface region between Notch1 and DLL4 proteins. EGR domain is colored with green, DLL4 is colored with blue. Calcium ions are represented as gray spheres. Mutated residue in 816 position is marked in red color.

**Figure 2:**
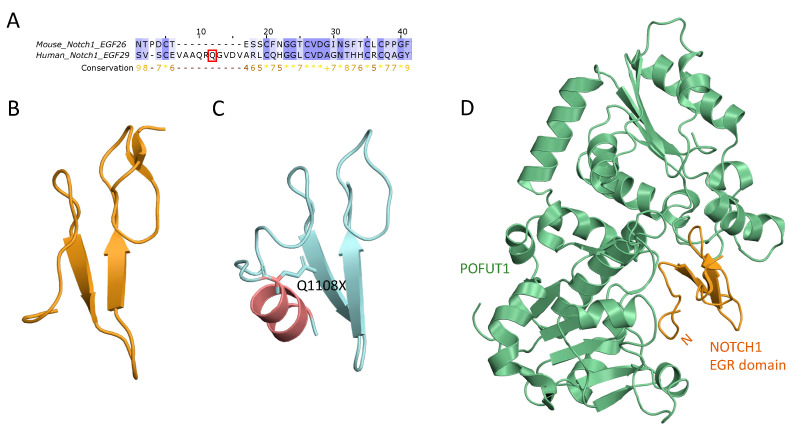
The Notch1 EGR-like domain. (**A**). A local alignment of a mouse Notch1 EGF26 (PDB ID: 5ky4 chain B). with a human Notch1 EGF29. Conservation score is visualized under the alignment for each column. Residues matching the consensus sequence residue at a given position are colored dark blue, while those that do not match the consensus residue, but have a positive BLOSUM62 score [30], are colored light blue. Positions with conservation score equal to 10 are marked as ‘+’. Positions with all identical residues are marked as ‘asterisk’. Yellow arrows indicate β-strands, red barrel indicates α-helix (**B**) A crystal structure of mouse Notch1 EGR-domain (PDB ID: 5ky4). (**C**) Alphafold2 model of a human Notch1 EGR-domain. (**D**). Mouse POFUT1 (green) in complex with mouse Notch1 EGF26 (orange) [31]. Orange letter ‘N’ means N-terminal part of an EGR-like domain.

**Figure 3:**
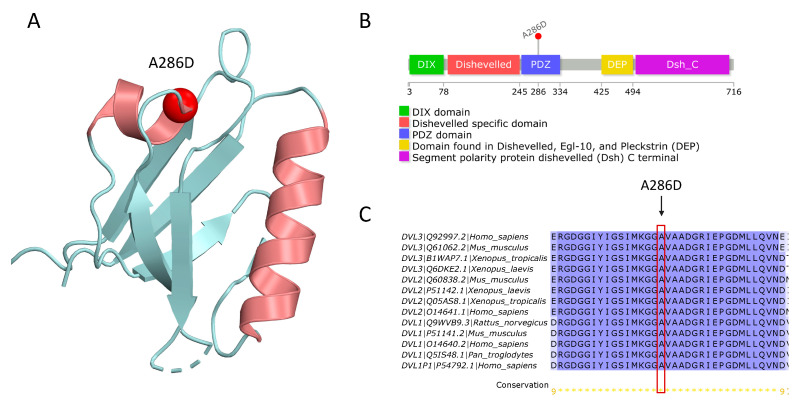
A human DVL3 protein. (**A**) X-ray structure of the PDZ domain of DVL3 (PDB ID: 6zbq). Mutated residue in 286 position is marked as a sphere in red color. (**B**) Domain organization of the human DVL3 protein sequence performed using the Uniprot database. Variant p.Ala286Asp is mapped to the protein structure. (**C**) A local alignment of a human PDZ domain with orthologues. Conservation score is visualized as in Figure 2A.

In addition to ACMG interpretation, pathogenic/benign status of variants was assessed using three prediction tools: VEST4, REVEL, and ClinPred (Table 7). Variants p.Asn816Asp and p.Gln1108Ter in Notch1, and p.Ala286Asp in DVL3 were classified as pathogenic by all tools. In contrast, variants p.Pro679Ser in DVL2 and p.Gln186His in DTX3L were not damaging, and two variants, p.Val571Ile (DTX4) and p.Arg83His (DTX1), had conflicting interpretation (Table 7). Variants p.Val571Ile (DTX4), p.Arg83His (DTX1), p.Gln1108Ter (NOTCH1), p.Ala286Asp (NOTCH1), and p.Ala286Asp (DVL3) are located in highly conserved positions in the protein (their Cs scores ≥ 5). Of note, in Notch1 protein, missense variants p.Asn816Asp and p.Gln1108Ter are located in the EGR21 and EGR29 domains, respectively, and can participate in several interprotein interactions (Figure 1A). According to the structural modeling, p.Asn816Asp participates in the NOTCH1/DLL4 interaction in the interface between two proteins and can be important in protein binding (Figure 1B,C). The terminated variant in 1108 NOTCH1 position was found in EGF29 (p.Gln1108Ter), which has high similarity with mouse Notch1 EGF26 (Figure 2A,B). Residue Gln is located in the 1108 position, which is absent in the mouse EGF26 domain. According to the Alphafold structure of the NOTCH1 EGF29, region 1100–1143 forms an alpha helix and may be important in binding with the POFUT1 protein (Figure 2C). The terminated mutation in 1108 may be crucial in forming the alpha helix in EGF29 (Figure 2D). In a human DVL3 protein, missense variant p.Ala286Asp was found in a PDZ domain (Figure 3). PDZ is a conserved domain among DVL proteins through which DVL binds to the membrane-bound receptor Frizzled and to other signal transduction molecules in the cytoplasm [27]. The PDZ domain consists of 89 amino acids comprising residues 245–334 which fold into two alpha-helices and six beta-strands exposing a distinct peptide-binding groove [28] (Figure 3A). Variant p.Ala286Asp is located in an alpha helix near a binding pocket which is formed by hydrophobic residues Phe259, Leu260, Gly261, Ile262, Ser263, Ile264, and Val316. Replacement of the highly conserved hydrophobic alanine in the 286 position by the polar asparagine may influence the recognition and binding affinity of the DVL PDZ domain and its binding partners.

To sum up, target sequencing of 20 NOTCH-signaling-related genes allowed us to identify rare genetic variants in the NOTCH1, DVL2, DVL3, DTX1, DTX3L and DTX4 genes with structural and functional impact on the NOTCH1 structure and on protein–protein interactions. Of the variants identified, two variants in NOTCH1 and a variant in DVL3 demonstrated prominent structural damaging effect and can potentially be associated with the development of aortic valve pathology.

## 4. Discussion

CAVD remains one of the most common heart diseases [32], with different genetic mechanisms for initiating aortic valve calcification in patients with BAV and TAV [33]. A number of studies support the association of variants in the NOTCH1 gene not only with BAV but with other cardiovascular abnormalities such as ascending aortic dilation and aortic coarctation [34,35]. Currently, the only effective treatment for severe aortic valve calcification is surgical aortic valve replacement [36], which, in turn, carries certain operational risks and places a heavy socioeconomic burden on the health care system [37]. In our study, we included 59 patients with BAV and 31 patients with TAV. According to a previous publication, the population is dominated by men with AS, while in our study, the groups of men and women were comparable [38]. The mean age of patients with AS and BAV was lower than that of patients with AS and TAV, which is consistent with the available data that patients with BAV were younger than patients with TAV [39]. One of the known risk factors for the development of aortic valve calcinosis is high blood pressure [37]. In our analysis, we found that patients with CAVD and TAV had significantly higher systolic and diastolic blood pressure values than patients with BAV, which is consistent with another publication confirming differences in the risk factors and pathogenesis of AS depending on valve morphology [40,41]. Patients with TAV had higher levels of C-reactive protein, which is consistent with the results of previously published studies and may be explained by the fact that patients with TAV are older and have more comorbid pathologies than patients with BAV [36,42]. However, we did not find significant differences in the incidence of chronic heart failure and coronary heart disease depending on the morphology of the valves, which is not fully consistent with the publication data, and this may be explained by the small size of the group. [2,3]. It was also found that carrying the T allele of the rs3812603 genotype of the NOTCH1 and the T allele of the rs73185723 genotype of the DLV3 was associated with higher blood pressure values in patients with CAVD. Some studies have described a relationship between aortic valve morphology, specifically the BAV, and the incidence of aortic dilation [43,44,45]. We found that carrying the C allele of the rs3812603 genotype of the NOTCH1 in patients with BAV was associated with larger aortic sinus.

The high population prevalence and great clinical significance of CAVD constantly initiate a variety of genetic and molecular studies on the process of aortic valve calcification. This research became even more significant and intense with the development of biological therapies, recognition of the role of inflammation in CAVD and new challenges in the fields of unticalcification drugs [46,47,48,49,50]. This triggered a new round of molecular studies linking the biochemical background (apoprotein(a), apoB, LDL-C, triglyceride level), environmental and acquired factors (smoking, obesity) and genetic factors. The latter became extensively explored due to GWAS and next-generation sequencing technologies leading to the description of several key polygenic risk factor and monogenic causes and CAVD and bicuspid aortic valve. Among these genes and pathways is the Notch1 signaling cascade, described in connection to congenital heart disorders, bicuspid aortic valve and aortic aneurhysma infamilial cases of congenital heart and valve malformations. Later, several common genetic variants from the Notch1 cascade were confirmed to be related to CAVD, underlining the role of Notch1 cascade and Notch1-related genes in aortic arch and valve pathology [8,51,52,53,54]. In addition, several loci were demonstrated and replicated in genome-wide association studies, with an expression effect on the inflammatory genes and Notch-signaling associate genes according to GTEx database [55,56]. In our study, we further confirmed the role of Notch signaling in the development of aortic valve pathology and identified two common genetic variants and several rare amino acid substitutions in association with aortic calcification. The structural modeling of the identified variants revealed that at least three of them (Asn816Asp, Gln1108Ter in *NOTCH1* and in Ala286Asp *DVL3*) had a prominent effect on protein structure and function. We further identified two common variants (rs3812603 in *NOTCH1* intron and—rs73185723 in *DVL3*) in association with CAVD, which further underlined the importance of this gene in relation to aortic disorders. In addition, rs3812603 identified in *NOTCH1* seems to be the only protective variant in relation to CAVD development to date. This notion needs further validation and replication on bigger cohorts; however, the identification of potentially protective *NOTCH1* variants can initiate a new round of research focused on Notch-signaling genes. In this regard, it is important to note that eQTL analysis using GTEx demonstrated the link of rs3812603 with the expression *DNLZ*, and of rs73185723 with the expression of *HSP90AA5P*. For both, loci are in connection with the regulation of heat-shock-protein-related genes of smooth muscle cells and, potentially, can modify their chaperon activity [57]. In addition, DVL3 is able interfere with TAP/TAZ signaling and through nuclear translocation lead to upregulation of osteogenesis-associated genes independent of canonical Wnt/β-catenin signaling [58]. In addition, *DVL3* is known to play an essential role in left ventricular outfloor tract development. The association of this gene’s rare and common variants with CAVD supports its potential importance for inherited and acquired aortic valve disorders and cardiac outfloor tract pathologies. Taking into account the lower systolic and diastolic blood pressure in carriers of C allele of rs73185723 in the *DVL3* gene, we can assume that the carriers of these variants are more prone to aortic valve calcification independently of other CAVD risk factors and of valve morphology. The molecular processes underlying cardiac valve development include cell differentiation, proliferation, apoptosis and migration, as well as organization of the extracellular matrix. Any minor change in the molecular signals regulating cell differentiation, proliferation, apoptosis and migration of aortic valve cells, as well as the organization of the extracellular matrix due to tiny modulation of Notch1-Wnt-signaling, can cause congenital heart valve defects, including BAV, that will directly affect the predisposition to CAVD [9].

## 5. Conclusions

We confirmed the prominent role of Notch-signaling pathway genes (*NOTCH1* and *DVL3*) in the development of CAVD and, for the first time, suggested the protective role of rs3812603 in AS development regardless of the presence or absence of BAV.

## 6. Limitations

This study has several important limitations, such as the small number of patients included in this study (less than 100 patients), and the unavailability of the genetic material from the relatives to uncover the pathogenic effect of the genetic variants. In addition, patients with mild and moderate severity of AS, connective tissue diseases, chronic rheumatic disease and infective endocarditis were also excluded from the study group.

## Figures and Tables

**Table 1:** Demographic and clinical characteristics of CAVD patients depending on valve morphology.

Characteristics	AS Group with BAV Me (Q1;Q3) (n = 59)	AS Group with TAV Me (Q1;Q3) (n = 31)	*p* Value
Age, years	56 (52;61)	61 (60;63)	0.00004
Gender, m/f	1.36:1	1:1.2	0.26
Arterial hypertension, n (%)	11 (18.6)	9 (29.03)	0.39
Chronic heart failure, n (%)	19 (32.2)	12 (38.7)	0.54
Coronary heart disease, n (%)	12 (20.3)	11 (3.5)	0.12
Systolic BP, mmHg	160 (140;180)	180 (170;200)	0.02
Diastolic BP, mmHg	90 (85;100)	100 (100;110)	0.02
Systolic BPof, mmHg	130 (120;150)	130 (120;156)	0.27
Diastolic BPof, mmHg	80 (74;90)	80 (70;90)	0.79
Aortic sinus, mm	34 (32.3;40)	35 (33;39)	0.66
Ascending aorta, mm	39 (35;43)	37.5 (35;40)	0.36
Mean pressure gradient, mmHg	49.7 (44;70)	50 (40;62)	0.37
Vmax, m/s	4.7 (4.24;5.18)	4.6 (4.2;5)	0.42
AVA, cm^2^	0.8 (0.7;0.9)	0.9 (0.7;1)	0.21
Hemoglobin, g/dL	138 (129;150)	136 (132;145)	0.43
Red blood cell count, ×10^6^ cells/micro-L	4.65 (4.3;5.1)	4.7 (4.47;4.84)	0.75
White blood cell count, ×10^3^ cells/micro-L	6.4 (5.7;7.6)	6.6 (5.6;7.5)	0.96
C-reactive protein, mg/dL	1.56 (0.85;2.77)	2.01 (1.49;3.48)	0.02
Blood glucose level, mmol/L	5.5 (5;6.15)	5.96 (5.2;6.46)	0.08
Blood creatinine level, mmol/L	79 (71;90)	75.5 (65;87)	0.25
Total cholesterol, mmol/L	5.7 (4.72;6.48)	5.12 (4;5.63)	0.03
Triglicerides, mmol/L	1.36 (0.99;2.07)	1.08 (0.91;1.62)	0.39
Low density lipoprotein, mmol/L	3.78 (2.64;4.24)	3.28 (2.3;4.4)	0.54
High-density lipoproteins, mmol/L	1.22 (0.98;1.5)	1.17 (1;1.59)	0.87

BP—blood pressure; BPof—office blood pressure; Vmax—peak aortic jet velocity; AVA: aortic valve area.

**Table 2:** Clinical characteristics of CAVD patients depending on rs3812603 genotype.

Characteristics	rs3812603		*p* Value
	Allele T Me (Q1;Q3) (n = 25)	Allele C Me (Q1;Q3) (n = 51)	
Age, years	57 (53;61)	60 (53;63)	0.22
Gender, m : f	1:1.08	1.2:1	0.57
BAV, n (%)	18 (72)	33 (64.7)	0.52
Arterial hypertension, n (%)	17 (68)	45 (88.2)	0.07
Chronic heart failure, n (%)	6 (24)	20 (39.2)	0.29
Coronary heart disease, n (%)	7 (28)	13 (25.5)	0.96
Systolic BP, mmHg	160 (140;180)	180 (150;190)	0.02
Diastolic BP, mmHg	100 (80;100)	100 (90;100)	0.15
Systolic BPof, mmHg	130 (120;140)	135 (120;150)	0.19
Diastolic BPof, mmHg	80 (78;90)	80 (70;90)	0.52
Aortic sinus, mm	33 (32;36)	35 (33;40)	0.07
Ascending aorta, mm	38 (34;45)	39 (36;43)	0.89
Aortic maximum, mm	38 (35;42)	40 (36;43)	0.53
Mean pressure gradient, mmHg	47.9 (43.5;64)	54.5 (44;72)	0.44
Vmax, m/s	4.42 (4.2;4.96)	4.88 (4.34;5.2)	0.20
AVA, cm^2^	0.83 (0.7;0.96)	0.85 (0.7;0.9)	0.75

BAV—bicuspid aortic valves; BP—blood pressure; BPof—office blood pressure; Vmax—peak aortic jet velocity; AVA: aortic valve area.

**Table 3:** Clinical characteristics of BAV CAVD patients depending on rs3812603 genotype.

Characteristics	rs3812603		*p* Value
	Allele T Me (Q1;Q3) (n = 18)	Allele C Me (Q1;Q3) (n = 33)	
Age, years	55.5 (50;59)	56 (52;61)	0.441054
Gender, m : f	1:1.25	2:1	0.1071
Arterial hypertension, n (%)	12 (66.7)	28 (84.8)	0.1254
Chronic heart failure, n (%)	7 (38.9)	14 (42.4)	0.5230
Coronary heart disease, n (%)	5 (27.8)	6 (18.2)	0.4398
Systolic BP, mmHg	160 (140:170)	170 (140;185)	0.141425
Diastolic BP, mmHg	90 (80;100)	92.5 (90;100)	0.170381
Systolic BPof, mmHg	129 (120;150)	130 (120;150)	0.944091
Diastolic BPof, mmHg	80.5 (80;90)	80 (75;85)	0.233399
Aortic sinus, mm	33 (32;35)	36 (33;40)	0.028920
Ascending aorta, mm	39 (35;45)	40 (36;44)	0.932005
Aortic maximum, mm	40 (36;45)	41(36;44)	0.774599
Mean pressure gradient, mmHg	48.5 (43;70)	55(44;72)	0.839622
Vmax, m/s	4.6 (4.1;5.4)	4.9 (4.38;5.2)	0.831224
AVA, cm^2^	0.7 (0.6;0.8)	0.85 (0.7;0.9)	0.146324

BP—blood pressure; BPof—office blood pressure; Vmax—peak aortic jet velocity; AVA: aortic valve area.

**Table 4:** Clinical characteristics of CAVD patients depending on rs73185723 genotype.

Characteristics	rs73185723		*p* Value
	Allele T Me (Q1;Q3) (n = 32)	Allele C Me (Q1;Q3) (n = 44)	
Age, years	61 (55;64)	59 (53;62)	0.1612173
Gender, m/f	1:0.9	1:1	0.8906
Arterial hypertension, n (%)	27 (84.4)	34 (77.3)	0.4387
Chronic heart failure, n (%)	15 (46.9)	15 (34.1)	0.2164
Coronary heart disease, n (%)	7 (21.9)	24 (54.5)	0.5219
Systolic BP, mmHg	180 (160;200)	160 (140;180)	0.006908
Diastolic BP, mmHg	100 (90;100)	90 (85;100)	0.036068
Systolic BPof, mmHg	130 (120;150)	130 (120;140)	0.136841
Diastolic BPof, mmHg	80 (74;90)	80 (70;87.5)	0.611371
Aortic sinus, mm	35 (33;40)	34 (32;39)	0.478722
Ascending aorta, mm	39 (37;42)	38 (34;42)	0.245006
Aortic maximum, mm	40 (37;42)	38.5 (34.5;42.5)	0.406663
Mean pressure gradient, mmHg	53.5 (46;64)	49 (43;66)	0.714627
Vmax, m/s	4.8 (4.1;5.1)	4.7 (4.2;4.9)	0.256888
AVA, cm^2^	0.83 (0.65;0.93)	0.85 (0.7;1)	0.498415

BP—blood pressure; BPof—office blood pressure; Vmax—peak aortic jet velocity; AVA: aortic valve area.

**Table 5:** Clinical characteristics of BAV CAVD patients depending on rs73185723 genotype.

Characteristics	rs73185723		*p* Value
	Allele T Me (Q1;Q3) (n = 20)	Allele C Me (Q1;Q3) (n = 27)	
Age, years	59 (52.5;64)	55 (50;60)	0.1735001
Gender, m/f	1.2:1	1.1:1	0.8307
Arterial hypertension, n (%)	16 (80)	19 (70.4)	0.6816
Chronic heart failure, n (%)	11 (55)	8 (29.6)	0.0797
Coronary heart disease, n (%)	4 (20)	3 (11.1)	0.6658
Systolic BP, mmHg	180 (150;200)	145 (140;165)	0.004804
Diastolic BP, mmHg	100 (90;100)	90 (80;90)	0.022784
Systolic BPof, mmHg	140 (120;150)	125 (120;140)	0.056344
Diastolic BPof, mmHg	80 (77;90)	80 (70;85)	0.320321
Aortic sinus, mm	35.5 (33;40.5)	33.5 (32;37)	0.181866
Ascending aorta, mm	39 (37;42)	39 (33;45)	0.760475
Aortic maximum, mm	40 (37;42.5)	39 (33;46)	0.586622
Mean pressure gradient, mmHg	49.7 (42;63)	51 (43.5;70.8)	0.684966
Vmax, m/s	4.70 (4.1;5.14)	4.68 (4.4;5)	0.990928
AVA, cm^2^	0.85 (0.7; 0.96)	0.75 (0.7;0.9)	0.603780

BP—blood pressure; BPof—office blood pressure; Vmax—peak aortic jet velocity; AVA: aortic valve area.

**Table 6:** New and rare (<1:10,000) genetic variants in NOTCH-signaling related genes detected in patients with CAVD.

Patient	Gene	Chromosome Position (hg39) and Variant Nomenclature	rs MAF	ACMG Classification
227	DTX4	Chr11:59204760, NM_015177.2:c.1711G>A:p.V571I	rs376862310 0.00002	LB
189	NOTCH1	Chr9:136513042, NM_017617.5:c.2446A>G:p.N816D	rs1589064290 -	VUS
	DTX1	Chr12:113058440, NM_004416.3:c.248G>A: p.R83H	rs772474000 0.00002	VUS
180	DVL2	Chr7:7226041, NM_004422.3:c.2035C>T: p.P679S	rs147610025 0.00006	VUS
166	NOTCH1	Chr9: 136508235, NM_017617.5:c.3322C>T, pQ1108Ter	-	LP
004	DTX3L	Chr 3:122568647, NM_138287.3:c.558A>C:p.Q186H	rs1466715187 -	VUS
048	DVL3	Chr3:184166219, NM_004423.4:c.857C>Ap.A286D	rs1358353596 C>T -	VUS

The clinical significance of the variants was assessed according to ACMG guidelines. LB—likely benign, VUS—variant of unknown significance.

**Table 7:** Functional prediction of missense variants in proteins by sequence-based computational methods.

Gene	Uniprot	Protein Variant	Domain	Cs	VEST4	REVEL	ClinPred	SIFT	Mutation Taster	PROVEAN
DTX4	Q9Y2E6	p.Val571Ile	DTC	9	D	T	T	pathogenic	uncertain	benign moderated
NOTCH1	P46531	p.Asn816Asp	EGR-CA	3	D	D	D	benign moderated	uncertain	benign
DTX1	Q86Y01	p.Arg83His	WWE	11	D	T	D	uncertain	uncertain	uncertain
DVL2	O14641	p.Pro679Ser	Dsh-C	0	T	T	T	benign	uncertain	benign
NOTCH1	P46531	p.Gln1108Ter	EGR	5	D	D	D	-	uncertain	-
DTX3L	Q8TDB6	p.Gln186His	disorder	0	T	T	T	benign	benign	benign
DVL3	Q92997	p.Ala286Asp	PDZ	11	D	D	D	pathogenic	uncertain	pathogenic

UniProt ID: accession number of a protein in the Uniprot KB database encoded by gene; Domain: protein domain which contains missense variant. Domain abbreviation as in Uniprot database: DTC, Deltex C-terminal domain; EGR-CA, calcium-binding epidermal growth factor-like domain; WWE, the WWE domain is named after three of its conserved residues; Dsh-C, segment polarity protein dishevelled C-terminal; EGR, epidermal-growth-factor-like domain; PDZ, PDZ-domain, also known as discs-large homologous regions. Cs, conservation score of a mutated position in the alignment. For VEST4, REVEL, and ClinPred we used the following abbreviations: D—damaging; T—tolerated.

## Data Availability

The datasets generated and analyzed for this study can be requested from the corresponding author.

## References

[1] Osnabrugge R.L., Mylotte D., Head S.J., Van Mieghem N.M., Nkomo V.T., LeReun C.M., Bogers A.J., Piazza N., Kappetein A.P. (2013). Aortic stenosis in the elderly: Disease prevalence and number of candidates for transcatheter aortic valve replacement: A meta-analysis and modeling study. J. Am. Coll. Cardiol..

[2] Vahanian A., Beyersdorf F., Praz F., Milojevic M., Baldus S., Bauersachs J., Capodanno D., Conradi L., De Bonis M., De Paulis R. (2022). 2021 ESC/EACTS Guidelines for the management of valvular heart disease: Developed by the Task Force for the management of valvular heart disease of the European Society of Cardiology (ESC) and the European Association for Cardio-Thoracic Surgery (EACTS). Rev. Espanola Cardiol. (Engl. Ed.).

[3] Danielsen R., Aspelund T., Harris T.B., Gudnason V. (2014). The prevalence of aortic stenosis in the elderly in Iceland and predictions for the coming decades: The AGES–Reykjavík study. Int. J. Cardiol..

[4] Nkomo V.T., Gardin J.M., Skelton T.N., Gottdiener J.S., Scott C.G., Enriquez-Sarano M. (2006). Burden of valvular heart diseases: A population-based study. Lancet.

[5] Peeters F.E.C.M., Meex S.J.R., Dweck M.R., Aikawa E., Crijns H.J.G.M., Schurgers L.J., Kietselaer B.L.J.H. (2017). Calcific aortic valve stenosis: Hard disease in the heart: A biomolecular approach towards diagnosis and treatment. Eur. Heart J..

[6] Ferreira-González I., Pinar-Sopena J., Ribera A., Marsal J.R., Cascant P., González-Alujas T., Evangelista A., Brotons C., Moral I., Permanyer-Miralda G. (2012). Prevalence of calcific aortic valve disease in the elderly and associated risk factors: A population-based study in a Mediterranean area. Eur. J. Prev. Cardiol..

[7] Coffey S., Cairns B.J., Iung B. (2015). The modern epidemiology of heart valve disease. Heart.

[8] Irtyuga O., Malashicheva A., Zhiduleva E., Freylikhman O., Rotar O., Bäck M., Tarnovskaya S., Kostareva A., Moiseeva O. (2017). NOTCH1 Mutations in Aortic Stenosis: Association with Osteoprotegerin/RANK/RANKL. BioMed Res. Int..

[9] Wang Y., Fang Y., Lu P., Wu B., Zhou B. (2021). NOTCH Signaling in Aortic Valve Development and Calcific Aortic Valve Disease. Front. Cardiovasc. Med..

[10] Irtyuga O.I., Zhiduleva E.Z., Dubrovskaya O.D., Moiseeva O.M. (2014). Concentration of Osteoprotegerin and RANKL in Blood Serum of Patients With Aortic Stenosis. Kardiologiia.

[11] Shikov A.E., Skitchenko R.K., Predeus A.V., Barbitoff Y.A. (2020). Phenome-wide functional dissection of pleiotropic effects highlights key molecular pathways for human complex traits. Sci. Rep..

[12] Lescroart F., Wang X., Lin X., Swedlund B., Gargouri S., Sànchez-Dànes A., Moignard V., Dubois C., Paulissen C., Kinston S. (2018). Defining the earliest step of cardiovascular lineage segregation by single-cell RNA-seq. Science.

[13] Stefansson H., Petursson H., Sigurdsson E., Steinthorsdottir V., Bjornsdottir S., Sigmundsson T., Ghosh S., Brynjolfsson J., Gunnarsdottir S., Ivarsson O. (2002). Neuregulin 1 and Susceptibility to Schizophrenia. Am. J. Hum. Genet..

[14] Wang Y., Wu B., Farrar E., Lui W., Lu P., Zhang D., Alfieri C.M., Mao K., Chu M., Yang D. (2015). Notch-Tnf signalling is required for development and homeostasis of arterial valves. Eur. Heart J..

[15] High F.A., Epstein J.A. (2008). The multifaceted role of Notch in cardiac development and disease. Nat. Rev. Genet..

[16] Limbourg F.P., Takeshita K., Radtke F., Bronson R.T., Chin M.T., Liao J.K. (2005). Essential role of endothelial Notch1 in angiogenesis. Circulation.

[17] Usoltsev D., Kolosov N., Rotar O., Loboda A., Boyarinova M., Moguchaya E., Kolesova E., Erina A., Tolkunova K., Rezapova V. (2023). Understanding Complex Trait Susceptibilities and Ethnical Diversity in a Sample of 4145 Russians Through Analysis of Clinical and Genetic Data. bioRxiv.

[18] Baumgartner H., Hung J., Bermejo J., Chambers J.B., Edvardsen T., Goldstein S., Lancellotti P., LeFevre M., Miller F., Otto C.M. (2016). Recommendations on the echocardiographic assessment of aortic valve stenosis: A focused update from the European Association of Cardiovascular Imaging and the American Society of Echocardiography. Eur. Heart. J. Cardiovasc. Imaging.

[19] Kostareva A., Kiselev A., Gudkova A., Frishman G., Ruepp A., Frishman D., Smolina N., Tarnovskaya S., Nilsson D., Zlotina A. (2016). Genetic Spectrum of Idiopathic Restrictive Cardiomyopathy Uncovered by Next-Generation Sequencing. PLoS ONE.

[20] Richards S., Aziz N., Bale S., Bick D., Das S., Gastier-Foster J., Grody W.W., Hegde M., Lyon E., Spector E. (2015). Standards and guidelines for the interpretation of sequence variants: A joint consensus recommendation of the American College of Medical Genetics and Genomics and the Association for Molecular Pathology. Genet. Med..

[21] UniProt Consortium (2015). UniProt: A hub for protein information. Nucleic Acids Res..

[22] Liu X., Li C., Mou C., Dong Y., Tu Y. (2020). dbNSFP v4: A comprehensive database of transcript-specific functional predictions and annotations for human nonsynonymous and splice-site SNVs. Genome Med..

[23] Altschul S.F., Gish W., Miller W., Myers E.W., Lipman D.J. (1990). Basic local alignment search tool. J. Mol. Biol..

[24] Notredame C., Higgins D.G., Heringa J. (2000). T-Coffee: A novel method for fast and accurate multiple sequence alignment. J. Mol. Biol..

[25] Waterhouse A.M., Procter J.B., Martin D.M.A., Clamp M., Barton G.J. (2009). Jalview Version 2—A multiple sequence alignment editor and analysis workbench. Bioinformatics.

[26] Berman H.M., Westbrook J., Feng Z., Gilliland G., Bhat T.N., Weissig H., Shindyalov I.N., Bourne P.E. (2000). The Protein Data Bank. Nucleic Acids Res..

[27] Wong H.-C., Bourdelas A., Krauss A., Lee H.-J., Shao Y., Wu D., Mlodzik M., Shi D.-L., Zheng J. (2003). Direct Binding of the PDZ Domain of Dishevelled to a Conserved Internal Sequence in the C-Terminal Region of Frizzled. Mol. Cell.

[28] Lee I., Choi S., Yun J.-H., Seo S.H., Choi S., Choi K.-Y., Lee W. (2017). Crystal structure of the PDZ domain of mouse Dishevelled 1 and its interaction with CXXC5. Biochem. Biophys. Res. Commun..

[29] Luca V.C., Jude K.M., Pierce N.W., Nachury M.V., Fischer S., Garcia K.C. (2015). Structural basis for Notch1 engagement of Delta-like 4. Science.

[30] Styczynski M.P., Jensen K.L., Rigoutsos I., Stephanopoulos G. (2008). BLOSUM62 miscalculations improve search performance. Nat. Biotechnol..

[31] Li Z., Han K., E Pak J., Satkunarajah M., Zhou D., Rini J.M. (2017). Recognition of EGF-like domains by the Notch-modifying O-fucosyltransferase POFUT1. Nat. Chem. Biol..

[32] Moncla L.-H.M., Briend M., Bossé Y., Mathieu P. (2023). Calcific aortic valve disease: Mechanisms, prevention and treatment. Nat. Rev. Cardiol..

[33] Kostina A., Shishkova A., Ignatieva E., Irtyuga O., Bogdanova M., Levchuk K., Golovkin A., Zhiduleva E., Uspenskiy V., Moiseeva O. (2017). Different Notch signaling in cells from calcified bicuspid and tricuspid aortic valves. J. Mol. Cell. Cardiol..

[34] Niaz T., Poterucha J.T., Johnson J.N., Craviari C., Nienaber T., Palfreeman J., Cetta F., Hagler D.J. (2016). Incidence, morphology, and progression of bicuspid aortic valve in pediatric and young adult subjects with coexisting congenital heart defects. Congenit. Heart Dis..

[35] Wang L., Wang L.M., Chen W., Chen X. (2016). Bicuspid Aortic Valve: A Review of its Genetics and Clinical Significance. J. Heart Valve Dis..

[36] Lindman B.R., Clavel M.-A., Mathieu P., Iung B., Lancellotti P., Otto C.M., Pibarot P. (2016). Calcific aortic stenosis. Nat. Rev. Dis. Prim..

[37] Yi B., Zeng W., Lv L., Hua P. (2021). Changing epidemiology of calcific aortic valve disease: 30-year trends of incidence, prevalence, and deaths across 204 countries and territories. Aging.

[38] Kong W.K., Bax J.J., Michelena H.I., Delgado V. (2020). Sex differences in bicuspid aortic valve disease. Prog. Cardiovasc. Dis..

[39] Çelik M., Milojevic M., Durko A.P., Oei F.B., Bogers A.J., Mahtab E.A. (2021). Differences in baseline characteristics and outcomes of bicuspid and tricuspid aortic valves in surgical aortic valve replacement. Eur. J. Cardio-Thorac. Surg..

[40] Ljungberg J., Johansson B., Engström K.G., Norberg M., Bergdahl I.A., Söderberg S. (2019). Arterial hypertension and diastolic blood pressure associate with aortic stenosis. Scand. Cardiovasc. J..

[41] Liakos C.I., Grassos C.A., Papadopoulos D.P., Dimitriadis K.S., Tsioufis C.P., Tousoulis D. (2017). Arterial hypertension and aortic valve stenosis: Shedding light on a common “liaison”. Hell. J. Cardiol..

[42] Song J., Zheng Q., Ma X., Zhang Q., Xu Z., Zou C., Wang Z. (2019). Predictive Roles of Neutrophil-to-Lymphocyte Ratio and C-Reactive Protein in Patients with Calcific Aortic Valve Disease. Int. Heart J..

[43] Della Corte A., Michelena H.I., Citarella A., Votta E., Piatti F., Lo Presti F., Ashurov R., Cipollaro M., Forte A. (2021). Risk Stratification in Bicuspid Aortic Valve Aortopathy: Emerging Evidence and Future Perspectives. Curr. Probl. Cardiol..

[44] Michelena H.I., Della Corte A., Prakash S.K., Milewicz D.M., Evangelista A., Enriquez-Sarano M. (2015). Bicuspid aortic valve aortopathy in adults: Incidence, etiology, and clinical significance. Int. J. Cardiol..

[45] Swahn E., Lekedal H., Engvall J., Nyström F.H., Jonasson L. (2023). Prevalence and determinants of dilated ascending aorta in a Swedish population: A case-control study. Eur. Heart J. Open.

[46] Combi Z., Potor L., Nagy P., Sikura K., Ditrói T., Jurányi E.P., Galambos K., Szerafin T., Gergely P., Whiteman M. (2023). Hydrogen sulfide as an anti-calcification stratagem in human aortic valve: Altered biogenesis and mitochondrial metabolism of H2S lead to H2S deficiency in calcific aortic valve disease. Redox Biol..

[47] de Oliveira Sá M.P.B., Cavalcanti L.R.P., Perazzo M., Gomes R.A.F., Clavel M.-A., Pibarot P., Biondi-Zoccai G., Zhigalov K., Weymann A., Ruhparwar A. (2020). Calcific Aortic Valve Stenosis and Atherosclerotic Calcification. Curr. Atheroscler. Rep..

[48] Zhou Z., Li Y., Jiang W., Wang Z. (2023). Molecular Mechanism of Calycosin Inhibited Vascular Calcification. Nutrients.

[49] Phadwal K., Tan X., Koo E., Zhu D., MacRae V.E. (2023). Metformin ameliorates valve interstitial cell calcification by promoting autophagic flux. Sci. Rep..

[50] Wei X., Shen Z., Zhu M., Fang M., Wang S., Zhang T., Zhang B., Yang X., Lv Z., Duan Y. (2023). The pterostilbene-dihydropyrazole derivative Ptd-1 ameliorates vascular calcification by regulating inflammation. Int. Immunopharmacol..

[51] Garg V. (2016). Notch Signaling in Aortic Valve Development and Disease. Etiology and Morphogenesis of Congenital Heart Disease.

[52] Garg V., Muth A.N., Ransom J.F., Schluterman M.K., Barnes R., King I.N., Grossfeld P.D., Srivastava D. (2005). Mutations in NOTCH1 cause aortic valve disease. Nature.

[53] Ma M., Li Z., Mohamed M.A., Liu L., Wei X. (2021). Aortic root aortopathy in bicuspid aortic valve associated with high genetic risk. BMC Cardiovasc. Disord..

[54] Freylikhman O., Tatarinova T., Smolina N., Zhuk S., Klyushina A., Kiselev A., Moiseeva O., Sjoberg G., Malashicheva A., Kostareva A. (2014). Variants in the *NOTCH1* Gene in Patients with Aortic Coarctation. Congenit. Heart Dis..

[55] Small A.M., Peloso G.M., Linefsky J., Aragam J., Galloway A., Tanukonda V., Wang L.-C., Yu Z., Selvaraj M.S., Farber-Eger E.H. (2023). Multiancestry Genome-Wide Association Study of Aortic Stenosis Identifies Multiple Novel Loci in the Million Veteran Program. Circulation.

[56] Chen H.Y., Dina C., Small A.M., Shaffer C.M., Levinson R.T., Helgadóttir A., Capoulade R., Munter H.M., Martinsson A., Cairns B.J. (2023). Dyslipidemia, inflammation, calcification, and adiposity in aortic stenosis: A genome-wide study. Eur. Heart J..

[57] Dores-Silva P., Minari K., Ramos C., Barbosa L., Borges J. (2013). Structural and stability studies of the human mtHsp70-escort protein 1: An essential mortalin co-chaperone. Int. J. Biol. Macromol..

[58] Wang L., Chennupati R., Jin Y.-J., Li R., Wang S., Günther S., Offermanns S. (2020). YAP/TAZ Are Required to Suppress Osteogenic Differentiation of Vascular Smooth Muscle Cells. iScience.

